# The Function of MAPK Cascades in Response to Various Stresses in Horticultural Plants

**DOI:** 10.3389/fpls.2020.00952

**Published:** 2020-07-31

**Authors:** Xiaowen He, Chuanzeng Wang, Haibo Wang, Linguang Li, Chen Wang

**Affiliations:** ^1^ Shandong Institute of Pomology, Taian, China; ^2^ Shandong Academy of Agricultural Sciences, Jinan, China; ^3^ State Key Laboratory of Crop Biology, College of Life Sciences, Shandong Agricultural University, Taian, China

**Keywords:** signal transduction, MAPK cascades, horticultural plant, biotic stress, abiotic stress

## Abstract

The mitogen-activated protein kinase (MAPK) cascade is a highly conserved signaling transduction module that transduces extracellular stimuli into intracellular responses in plants. Early studies of plant MAPKs focused on their functions in model plants. Based on the results of whole-genome sequencing, many MAPKs have been identified in horticultural plants, such as tomato and apple. Recent studies revealed that the MAPK cascade also plays crucial roles in the biotic and abiotic stress responses of horticultural plants. In this review, we summarize the composition and classification of MAPK cascades in horticultural plants and recent research on this cascade in responses to abiotic stresses (such as drought, extreme temperature and high salinity) and biotic stresses (such as pathogen infection). In addition, we discuss the most advanced research themes related to plant MAPK cascades, thus facilitating research on MAPK cascade functions in horticultural plants.

## Introduction

Horticultural plants, including fruits, vegetables, ornamental trees and flowers, are important economically valuable crops around the world. However, during plant growth and development, horticultural crops often suffer from a variety of stresses, including biotic stresses (e.g., diseases, pests) and abiotic stresses (e.g., drought, extreme temperature, high salinity), and these stresses seriously affect the quality and yield of these crops (Bai et al., 2018). In the process of resisting adverse stresses, plants have evolved sophisticated, complex and effective defense mechanisms, including signal perception, signal transduction, transcriptional regulation and immune responses, to reduce or avoid damage (Kissoudis et al., 2014). Studying the damage to plants caused by stresses and the response mechanisms of plants under stresses has become one of the focuses in plant stress resistance research.

Phosphorylation is a very important posttranslational modification (PTM) and the main method of signal transduction. The phosphorylation of proteins can transmit and amplify external signals by changing the expression of downstream genes and other biological processes (Wang et al., 2019). Protein kinases are a class of enzymes that catalyze the phosphorylation of related proteins, and the serine/threonine protein kinase family of mitogen-activated protein kinases (MAPKs) is one of the most widely studied gene families.

MAPKs are highly conserved signaling transduction modules and participate in many signal transduction processes through MAPK cascades. A typical MAPK cascade is composed of MAPK (MPK), MAPK kinase (MAPKK, MAP2K, MKK or MEK) and MAPK kinase kinase (MAPKKK, MAP3K or MEKK) (Ichimura et al., 2002; Rodriguez et al., 2010). In a classical MAPK signaling cascade, MAPKKK is activated by stimulated plasma membrane receptors and transmits signals downstream (Wang et al., 2014; Çakır and Kılıçkaya, 2015). MAPKKK activates MAPKK by phosphorylating the conserved S/T-XXXXX-S/T (S/T is serine/threonine, and X is an arbitrary amino acid) motif in MAPKK (Rodriguez et al., 2010). Subsequently, MAPKK activates MAPK by phosphorylating the TXY (T is threonine, Y is tyrosine, and X is any amino acid) motif in MAPK (Taj et al., 2010). Finally, MAPK activates downstream kinases, enzymes, transcription factors and other response factors and transmits extracellular environmental signals into cells (Zhang M. et al., 2018). Through stage-by-stage phosphorylation, the MAPK cascade can transmit and amplify signals to downstream proteins and activate the expression of resistance genes (Hamel et al., 2006). MAPKs regulate the expression of many genes through the phosphorylation of proteins, especially the phosphorylation of many transcription factors (Morris, 2001; Zhang and Klessig, 2001). The MAPK cascade plays important roles in mediating cell differentiation, cell development, hormonal activity, and abiotic and biotic stress responses (Komis et al., 2018). Increasing evidence indicates that genetic manipulation of the abundance or activity of some MAPK components can enhance tolerance to many stresses in crop plant species (Šamajová et al., 2013). In recent years, the function of MAPK cascades in horticultural plants has received widespread attention. This review summarizes the composition and classification of MAPK cascades in horticultural plants and the roles of MAPK signaling pathways in biotic and abiotic stresses responses.

## Composition and Classification of MAPK Cascades in Horticultural Plants

Similar to model plants, the horticultural plant MAPK cascade also consists of three parts: MAPKKK, MAPKK and MAPK. By analyzing the genomes of various plants, the number of MAPKKKs was found to be the highest among the three superfamilies in the MAPK cascade, followed by MAPK and finally MAPKK (Xu and Zhang, 2015). A total of 80 *MAPKKKs*, 10 *MAPKKs* and 20 *MAPKs* have been reported in *Arabidopsis thaliana* (Colcombet and Hirt, 2008). Due to the development of whole-genome sequencing technology, many MAPK cascade components have been identified in horticultural plants. Eighty-nine *MAPKKKs*, five *MAPKKs* and 16 *MAPKs* can be found in tomato (*Solanum lycopersicum*) (Kong et al., 2012; Wu et al., 2014). Recent studies have demonstrated that 120 *MAPKKKs*, 9 *MAPKKs* and 26 *MAPKs* are present in the apple genome (Zhang et al., 2013; Sun et al., 2017). The cucumber (*Cucumis sativus* L.) genome-sequencing project discovered 59 *MAPKKKs*, six *MAPKKs* and 14 *MAPKs* (Wang et al., 2015). Twelve *FvMAPKs*, seven *FvMAPKKs* and 73 *FvMAPKKKs* were verified from the recently published strawberry (*Fragaria vesca*) genome (Shulaev et al., 2011; Zhou et al., 2017). The grapevine (*Vitis vinifera*) genome contains 14 *MAPKs*, five *MAPKKs* and 62 *MAPKKKs* (Çakır and Kılıçkaya, 2015).

**Figure 1:**
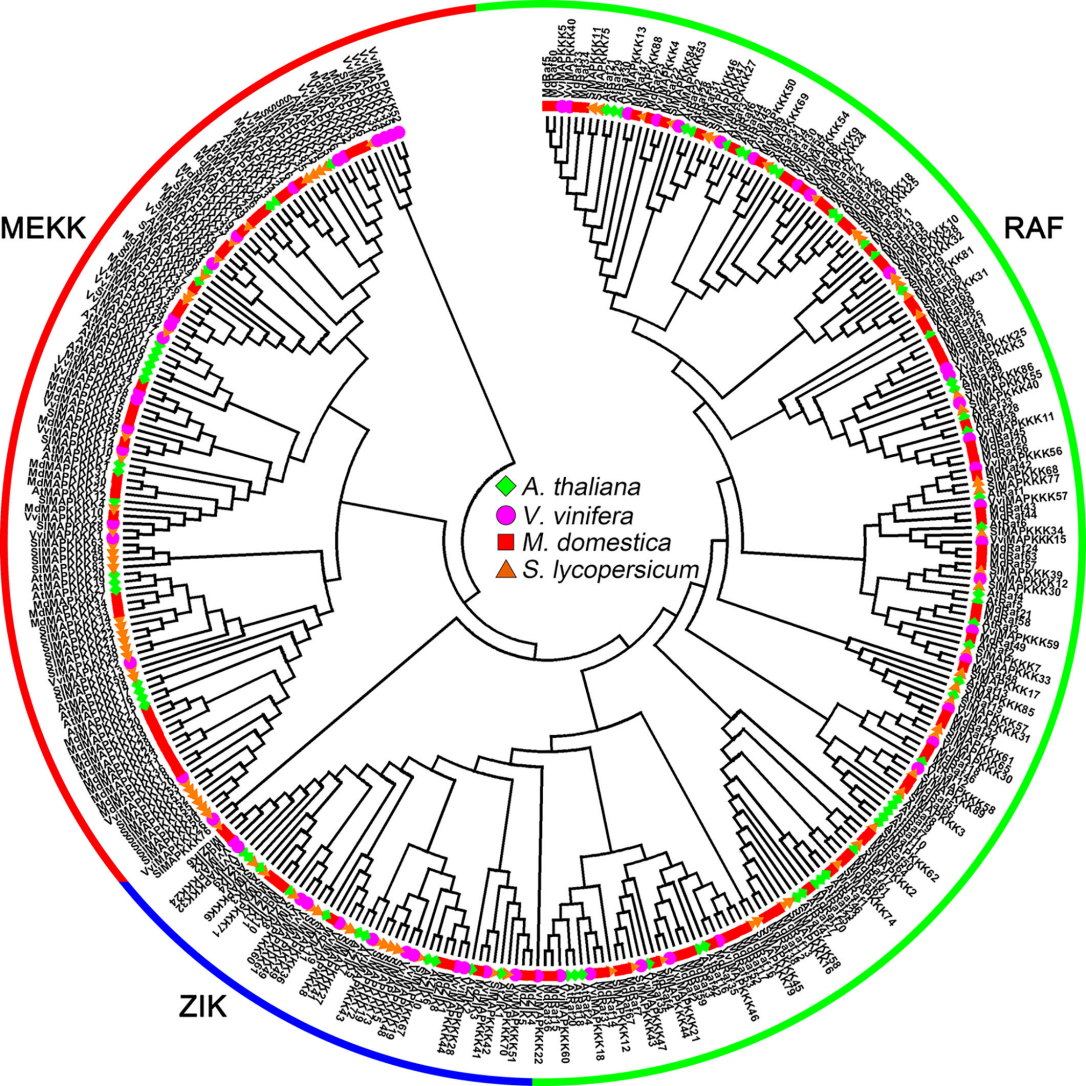
Phylogenetic analysis of MAPKKKs in various species. A total of 62 VviMAPKKKs from grapevine, 120 MdMAPKKKs from apple, 89 SlMAPKKKs from tomato and 78 AtMAPKKKs from *Arabidopsis* were used to create the neighbor-joining (NJ) tree using MEGA-X with 1,000 bootstraps.

According to characteristic sequence motifs, MAPK family genes (MAPKs, MAPKKs and MAPKKKs) have been divided into many subfamilies. Based on genome sequencing data, dendrogram analysis was used to classify MAPK family genes from three typical horticultural plants (tomato, grapevine and apple) using *Arabidopsis* as the standard. MAPKKK can be subdivided into three groups in higher plants: the MEKK subfamily, Raf subfamily and ZIK (ZR1-interacting kinase) subfamily (Rodriguez et al., 2010). In the members of the MEKK subfamily, a conserved G(T/S)Px(W/Y/F)MAPEV kinase domain can be found; most ZIK subfamily proteins have the GTPEFMAPE(L/V)Y domain, while Raf subfamily members have the GTxx(W/Y)MAPE domain (Jonak et al., 2002). The Raf and ZIK subfamily proteins have a C-terminal kinase domain (KD) and a long N-terminal regulatory domain (RD) that might function in scaffolding to recruit MAPKKs and MAPKs (Ichimura et al., 2002; Rodriguez et al., 2010). MEKK subfamily members have a less conserved protein structure compared with the ZIK subfamily and Raf subfamily (Rao et al., 2010; Rodriguez et al., 2010). In the tomato genome, 89 putative *MAPKKKs* have been identified, including 33 MEKK subfamily members, 16 ZIK subfamily members, and 40 Raf subfamily members (**Figure 1**) (Wu et al., 2014). Among the 62 *MAPKKKs* identified in grapevine, 21 *VviMAPKKKs* were in the MEKK subfamily, only 12 *VviMAPKKKs* belonged to the ZIK subfamily, and 29 *VviMAPKKKs* were grouped in the Raf subfamily (**Figure 1**) (Çakır and Kılıçkaya, 2015). In apple, a total of 72 putative *MdMAPKKKs* were identified in the Raf subfamily, 11 in the ZIK subfamily and 37 in the MEKK subfamily (**Figure 1**) (Sun et al., 2017).

**Figure 2:**
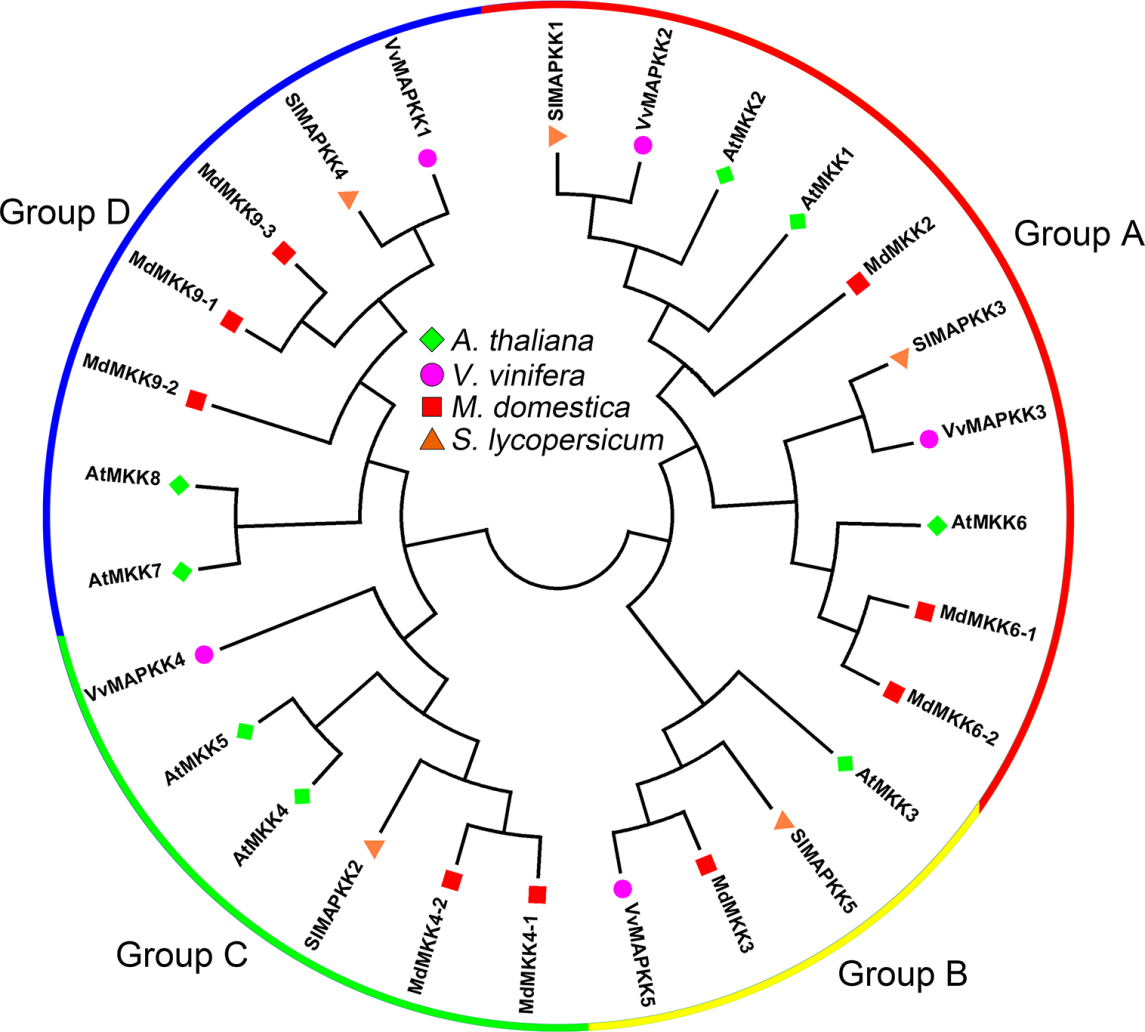
Phylogenetic analysis of MAPKKs in various species. A total of 5 VviMAPKKs from grapevine, 9 MdMAPKKs from apple, 5 SlMAPKKs from tomato, and eight AtMAPKKs from *Arabidopsis* were used to create the neighbor-joining (NJ) tree using MEGA-X with 1,000 bootstraps. Four clades were labeled as Group A, Group B, Group C and Group D.

MAPKKs can be divided into four groups, A, B, C and D, according to amino acid sequence analysis (Ichimura et al., 2002). Among the five tomato *MAPKKs*, *SlMAPKK1* and *SlMAPKK3* belong to group A, *SlMAPKK5* belongs to group B, *SlMAPKK2* belongs to group C, and *SlMAPKK4* belongs to group D (**Figure 2**) (Wu et al., 2014). For grapevine *MAPKKs*, *VvMKK2* and *VvMKK3* were highly homologous with group A *MAPKKs* (*AtMKK1*, *AtMKK2* and *AtMKK6*) in *Arabidopsis*, *VvMKK5* was highly homologous with group B *MAPKKs* (*AtMKK3*) in *Arabidopsis*, *VvMKK4* was highly homologous with group C *MAPKKs* (*AtMKK4* and *AtMKK5*) in *Arabidopsis*, and *VvMKK1* was highly homologous with group D *MAPKKs* (*AtMKK8*) in *Arabidopsis* (**Figure 2**) (Çakır and Kılıçkaya, 2015). Among the 9 *MKKs* of apple, *MKK2*, *MKK6-1* and *MKK6-2* belong to group A, *MKK3* belongs to group B, *MKK4-1* and *MKK4-2* belong to group C, and *MKK9-1*, *MKK9-2* and *MKK9-3* belong to group D (**Figure 2**) (Zhang et al., 2013).

**Figure 3:**
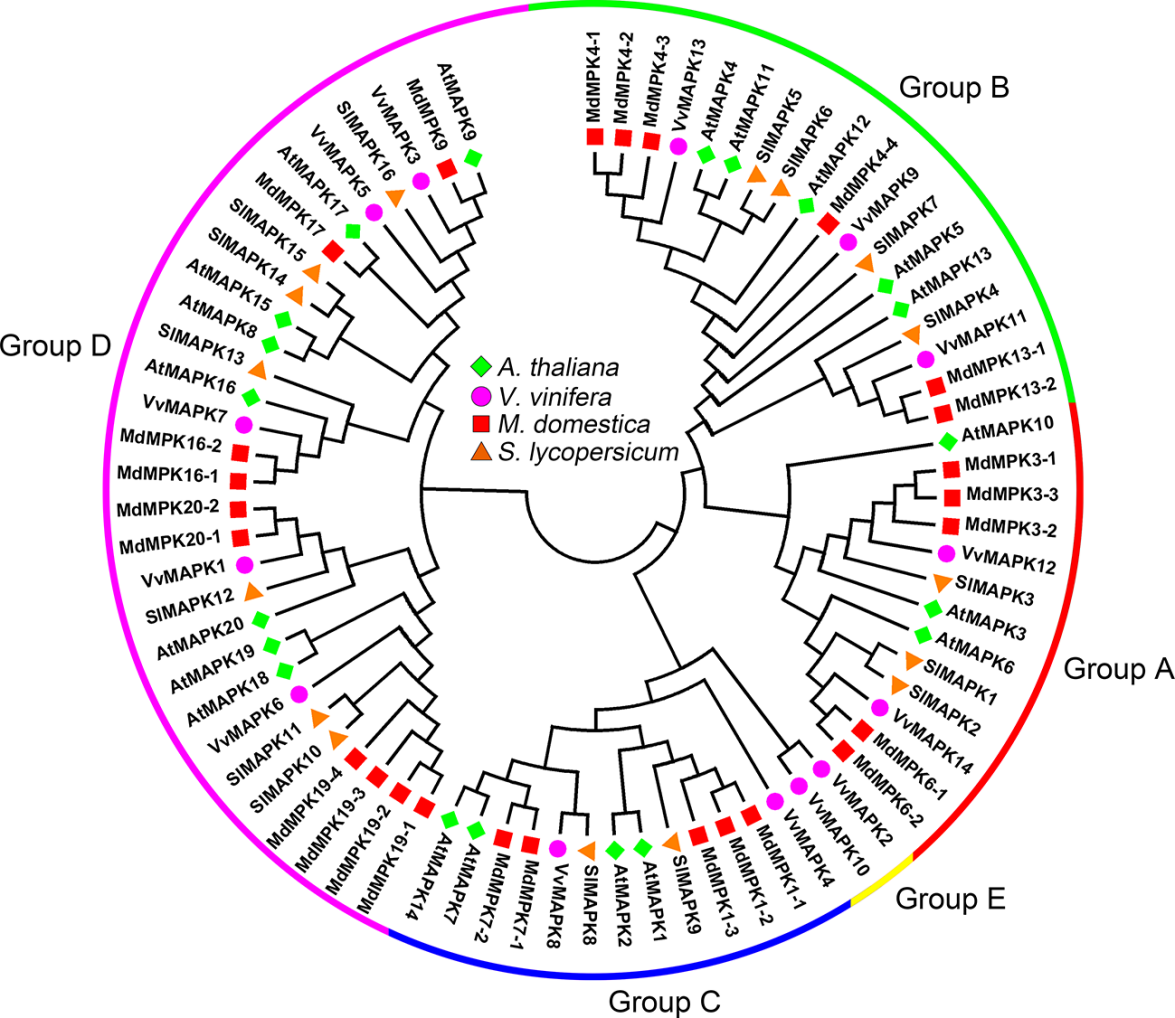
Phylogenetic analysis of MAPKs in various species. A total of 14 VviMAPKs from grapevine, 26 MdMAPKs from apple, 16 SlMAPKs from tomato and 20 AtMAPKs from *Arabidopsis* were used to create the neighbor-joining (NJ) tree using MEGA-X with 1,000 bootstraps.

According to the conserved T-X-Y motif phosphorylated by MAPKK, MAPKs can be divided into two subfamilies, with one containing TEY motifs and the other containing TDY motifs. Subfamilies containing TEY motifs can be classified into three groups based on their structural features and sequences (Jonak et al., 2002). In the tomato genome, three MAPK genes (*SlMAPK1*–*3*) belong to group A, four MAPK genes (*SlMAPK4*–*7*) belong to group B, two MAPK genes (*SlMAPK8*–*9*) belong to group C, and seven MAPK genes (*SlMAPK10*–*16*) belong to group D (**Figure 3**) (Kong et al., 2012). Although the grapevine genome contains fewer *MAPKs* than the *Arabidopsis* genome (20 *MAPKs*), the *VvMAPKs* have been divided into five subfamilies, which are different from those in other plant species (Çakır and Kılıçkaya, 2015). *VvMAPK12* and *VvMAPK14* are clustered in group A, *VvMAPK9*, *VvMAPK11*, and *VvMAPK13* belong to group B, *VvMAPK4* and *VvMAPK8* are clustered in group C, the group D MAPKs in grapevine include *VvMAPK1*, *VvMAPK3*, *VvMAPK5*, *VvMAPK6* and *VvMAPK7*, and *VvMAPK2* and *VvMAPK10* belong to group E, separate from the other groups (**Figure 3**) (Çakır and Kılıçkaya, 2015). The MAPK gene family in apple is by far the largest compared to the estimates for other plant species. The phylogenetic tree divided the MAPKs into four groups (groups A, B, C and D) of monophyletic clades. Groups A and C both contain five apple *MAPK* genes, followed by group B (6 genes), and group D constitutes the largest clade containing 10 *MdMAPKs* (**Figure 3**) (Zhang et al., 2013).

## The Function of MAPK Cascades in Responses to Abiotic Stresses in Horticultural Plants

Facing abiotic stresses, such as drought, extreme temperature, and salinity, plants have generated specific mechanisms that can activate secondary messenger-mediated signal transduction, regulate the expression of resistance genes and ultimately help plants adapt and survive under these adverse stresses (Zhu, 2016; Zandalinas et al., 2019). In horticultural plants, MAPK cascades participate in responses to numerous abiotic stresses, including drought, extreme temperature, salinity, ozone and UV irradiation (**Table 1**) (Ramani and Chelliah, 2007; Meng et al., 2014; Zhou et al., 2014; Yanagawa et al., 2016; Ji et al., 2017).

**Table 1:** Overview of MAPKs involved in different stress responses in different species.

Species	Gene Name	Response to Stress	Up/Down Regulated	References
*Actinidia Chinensis*	*AcMAPK4*	Salt Stress	up	Wang G. et al., 2018
	*AcMAPK5*	Salt Stress	up	Wang G. et al., 2018
	*AcMAPK9*	Salt Stress	up	Wang G. et al., 2018
	*AcMAPK12*	Salt Stress	up	Wang G. et al., 2018
*Brassica napus*	*BnMAPK1*	Response to Drought Stress		Weng et al., 2014
*Brassica rapa*	*BraMKK9*	*Plasmodiophora brassicae* infection	up	Piao et al., 2018
	*BraMPK1*	*Plasmodiophora brassicae* infection	up	Piao et al., 2018
	*BraMPK2*	*Plasmodiophora brassicae* infection	up	Piao et al., 2018
	*BraMPK5*	*Plasmodiophora brassicae* infection	up	Piao et al., 2018
	*BraMPK9*	*Plasmodiophora brassicae* infection	up	Piao et al., 2018
	*BraMPK19*	*Plasmodiophora brassicae* infection	up	Piao et al., 2018
	*BraMPK20*	*Plasmodiophora brassicae* infection	up	Piao et al., 2018
*Chrysanthemum morifolium*	*CmMPK1*	Low-temperature Stress	up	Song et al., 2018
	*CmMPK3.1*	Low-temperature Stress	up	Song et al., 2018
	*CmMPK3.2*	Low-temperature Stress	up	Song et al., 2018
	*CmMPK4.2*	Low-temperature Stress	up	Song et al., 2018
	*CmMPK6*	Low-temperature Stress	up	Song et al., 2018
	*CmMPK9.1*	Low-temperature Stress	up	Song et al., 2018
	*CmMPK9.2*	Low-temperature Stress	up	Song et al., 2018
	*CmMPK13*	Low-temperature Stress	up	Song et al., 2018
	*CmMPK16*	Low-temperature Stress	up	Song et al., 2018
	*CmMPK18*	Low-temperature Stress	up	Song et al., 2018
	*CmMPK1*	High-temperature Stress	down	Song et al., 2018
	*CmMPK9.1*	High-temperature Stress	down	Song et al., 2018
	*CmMPK9.2*	High-temperature Stress	down	Song et al., 2018
	*CmMPK16*	High-temperature Stress	down	Song et al., 2018
	*CmMPK18*	High-temperature Stress	down	Song et al., 2018
	*CmMPK4.2*	Salt and Drought Stresses	up	Song et al., 2018
	*CmMPK13*	Salt and Drought Stresses	up	Song et al., 2018
	*CmMKK2*	Salt and Drought Stresses	up	Song et al., 2018
	*CmMKK4*	Salt and Drought Stresses	up	Song et al., 2018
*Citrus sinensis*	*CsMAPK1*	Response to *Xanthomonas citri* Infection		de Oliveira et al., 2013
		Response to *Xanthomonas aurantifolii* Infection	
*Cucumis sativus*	*CsMKK4*	High-temperature Stress	up	Wang et al., 2015
*Fragaria vesca*	*FvMAPK5*	Drought Stress	up	Zhou et al., 2017
	*FvMAPK8*	Drought Stress	up	Zhou et al., 2017
	*FvMAPK3*	High-temperature Stress	up	Zhou et al., 2017
	*FvMAPKK1*	High-temperature Stress	up	Zhou et al., 2017
	*FvMAPKK3*	High-temperature Stress	up	Zhou et al., 2017
	*FvMAPKK6*	High-temperature Stress	up	Zhou et al., 2017
	*FvMAPKK7*	High-temperature Stress	up	Zhou et al., 2017
	*FvMAPK5*	Salt Stress	up	Zhou et al., 2017
	*FvMAPK9*	Salt Stress	up	Zhou et al., 2017
	*FvMAPK10*	Salt Stress	up	Zhou et al., 2017
	*FvMAPK11*	Salt Stress	up	Zhou et al., 2017
	*FvMAPK12*	Salt Stress	up	Zhou et al., 2017
	*FvMAPKK1*	Salt Stress	up	Zhou et al., 2017
	*FvMAPKK3*	Salt Stress	up	Zhou et al., 2017
	*FvMAPKK5*	Salt Stress	up	Zhou et al., 2017
*Malus*	*MaMAPK*	Drought Stress	up	Peng et al., 2006
	*MdRaf5*	Respnose to Drought Stress		Sun et al., 2017
*Manihot esculenta*	*MeMAPK4*	Salt Stress	down	Yan et al., 2016
	*MeMAPK16*	Salt Stress	down	Yan et al., 2016
	*MeMAPK17*	Salt Stress	down	Yan et al., 2016
	*MeMAPK19*	Salt Stress	down	Yan et al., 2016
	*MeMAPK1*	Salt Stress	up	Yan et al., 2016
*Moraceae morus*	*MnMAPK1*	Drought Stress	down	Wei et al., 2014
	*MnMAPK2*	Drought Stress	down	Wei et al., 2014
	*MnMAPK3*	Drought Stress	up	Wei et al., 2014
	*MnMAPK4*	Drought Stress	up	Wei et al., 2014
	*MnMAPK6*	Drought Stress	up	Wei et al., 2014
	*MnMAPK7*	Drought Stress	up	Wei et al., 2014
	*MnMAPK8*	Drought Stress	up	Wei et al., 2014
	*MnMAPK9*	Drought Stress	up	Wei et al., 2014
	*MnMAPK1*	High-temperature Stress	up	Wei et al., 2014
	*MnMAPK5*	High-temperature Stress	up	Wei et al., 2014
	*MnMAPK6*	High-temperature Stress	up	Wei et al., 2014
	*MnMAPK9*	High-temperature Stress	up	Wei et al., 2014
	*MnMAPK2*	High-temperature Stress	down	Wei et al., 2014
	*MnMAPK3*	High-temperature Stress	down	Wei et al., 2014
	*MnMAPK8*	High-temperature Stress	down	Wei et al., 2014
	*MnMAPK10*	High-temperature Stress	down	Wei et al., 2014
	*MnMAPK1*	Low-temperature Stress	up	Wei et al., 2014
	*MnMAPK5*	Low-temperature Stress	up	Wei et al., 2014
	*MnMAPK1*	Salt Stress	up	Wei et al., 2014
	*MnMAPK9*	Salt Stress	up	Wei et al., 2014
	*MnMAPK10*	Salt Stress	up	Wei et al., 2014
	*MnMAPK3*	Salt Stress	down	Wei et al., 2014
	*MnMAPK4*	Salt Stress	down	Wei et al., 2014
	*MnMAPK7*	Salt Stress	down	Wei et al., 2014
	*MnMAPK8*	Salt Stress	down	Wei et al., 2014
*Poncirus trifoliata*	*PtrMAPK*	Drought Stress	up	Huang et al., 2011
*Solanum lycopersicum*	*SlMPK1*	High-temperature Stress	down	Ding et al., 2018
	*SlMPK3*	Low-temperature Stress	up	Yu et al., 2015
	*SlMPK1*	Response to Herbivorous Insects Infection		Kandoth et al., 2007
	*SlMPK2*	Response to Herbivorous Insects Infection		Kandoth et al., 2007
	*SlMPK3*	Response to Herbivorous Insects Infection		Kandoth et al., 2007
	*SlMPK2*	Response to *Xanthomonas campestris* Infection		Melech-Bonfil and Sessa, 2011
	*SlMPK3*	Response to *Xanthomonas campestris* Infection		Mayrose et al., 2004
	*SlMKK2*	Response to *Pseudomonas syringae* Infection		Pedley and Martin, 2004
	*SlMKK4*	Response to *Pseudomonas syringae* Infection		Pedley and Martin, 2004
	*SlMAPKKKϵ*	Response to *Xanthomonas campestris* Infection		Melech-Bonfil and Sessa, 2010
		Response to *Pseudomonas syringae* Infection	
*Solanum tuberosm*	*StMEK2*	Response to *Phytophthora infestans* Infection		Wang H. et al., 2018
*Vitis vinifera*	*VviMAPKKK22*	Drought Stress	up	Wang et al., 2014
	*VviMAPKKK23*	Drought Stress	up	Wang et al., 2014
	*VviMAPKKK51*	Drought Stress	up	Wang et al., 2014
	*VviMAPKKK54*	Drought Stress	up	Wang et al., 2014
	*VviMAPKKK31*	*Erysiphe necator* Infection	up	Wang et al., 2014
	*VviMAPKKK32*	*Erysiphe necator* Infection	up	Wang et al., 2014
	*VviMAPKKK34*	*Erysiphe necator* Infection	up	Wang et al., 2014
	*VviMAPKKK38*	*Erysiphe necator* Infection	up	Wang et al., 2014
	*VviMAPKKK39*	*Erysiphe necator* Infection	up	Wang et al., 2014
	*VviMAPKKK46*	*Erysiphe necator* Infection	up	Wang et al., 2014
	*VviMAPKKK50*	*Erysiphe necator* Infection	up	Wang et al., 2014
	*VviMAPKKK4*	Powdery mildew Infection	down	Wang et al., 2014
	*VviMAPKKK51*	Powdery mildew Infection	down	Wang et al., 2014
	*VviMAPKKK54*	Powdery mildew Infection	down	Wang et al., 2014

### MAPK Cascades Involved in Regulating Drought Tolerance

Drought is a major environmental factor limiting the productivity and distribution of plants (Shi et al., 2011; Zhang H. et al., 2018). Horticultural plant roots are extensive and substantially affected by the soil moisture content, and their growth and products are therefore seriously influenced by drought stress. Analyzing the molecular mechanism of drought tolerance has great significance for breeding drought-tolerant varieties. The MAPK cascade plays an important role in the drought stress response in horticultural plants. Three species of *Malus* were used to study the expression of *MAPKs* in response to drought stress: *Malus hupehensis*, a drought-sensitive species; *Malus sieversii*, a drought-tolerant species; and *Malus micromalus*, a species with moderate tolerance (Peng et al., 2006). The highest expression level of *MaMAPK* (GenBank accession No. AF435805) was observed in *M. sieversii*, followed by *M. micromalus* and *M. hupehensis*. *MaMAPK* was dramatically induced after drought treatment for 1.5 h. This expression pattern was consistent with antioxidant enzyme activity in three apple species under drought treatment (Peng et al., 2006). Mounting evidence indicates that MAPK cascades play important roles in regulating drought tolerance in apple. In four apple species, *Malus hupehensis* (Pamp.) Rehd. var. *pinyiensis*, *Malus hupehensis* (Pamp.) Rehd. var. *taishanensis*, *Malus baccata* (L.) Borkn and *Malus sieversii* (Ledeb.) Roem, 12 *MAPKKKs* were highly regulated in leaves treated with 20% PEG for 3 h (Sun et al., 2017). Overexpression of *MdRaf5*, an MAPKKK Raf-like group gene, dramatically enhanced drought tolerance in transgenic *Arabidopsis* plants by reducing transpiration rates and stomatal apertures (Sun et al., 2017). A recent study reported that arbuscular mycorrhizal fungi (AMF) can enhance drought tolerance by using MAPK signals for interactions between AMF and their apple plant hosts (Huang et al., 2020). During drought, the expression levels of *MdMAPK16-2*, *MdMAPK17* and *MdMAPK20-1* were increased by 36.93%, 58.14% and 54.14%, respectively, compared to those in apple seedlings without AMF inoculation (Huang et al., 2020). Exclusive activation of some MAPK kinases occurs in drought-treated horticultural plants. *BnMAPK1* may be related to the response to drought stress in *Brassica napus*, and overexpression of *BnMAPK1* enhanced drought tolerance by increasing cell water retention and root activity (Weng et al., 2014). In cucumber, all examined *CsMAPKs* were initially downregulated for the first 2 days before they were significantly upregulated after drought treatment (Wang et al., 2015). In strawberry, *FvMAPK5* and *FvMAPK8* belong to group B, which contains well-characterized MAPK genes, including *AtMAPK3* and *AtMAPK6* (Zhou et al., 2017). Research has indicated that *FvMAPK5* and *FvMAPK8* are important for abiotic stress responses, with functions similar to those of *AtMAPK3* and *AtMAPK6* due to transcriptional activation by drought (Zhou et al., 2017). In trifoliate orange (*Poncirus trifoliata* L. Raf), transcript levels of *MAPKs* were increased by dehydration (Huang et al., 2011). Overexpression of *PtrMAPK* had a significant effect on the improvement of drought tolerance in transgenic tobacco plants. The morphological appearances of *PtrMAPK*-overexpression transgenic lines were better than those of WT plants as more leaves remained green in the transgenic lines (Huang et al., 2011). In mulberry, eight *MnMAPKs* were significantly induced by drought treatment, and two *MnMAPKs* (*MnMAPK1* and *MnMAPK2*) were significantly downregulated (Wei et al., 2014). Six *MnMAPKs* (*MnMAPK3*, *MnMAPK4*, *MnMAPK6*, *MnMAPK7*, *MnMAPK8* and *MnMAPK9*) showed positively regulated expression, particularly *MnMAPK7*, which had very high expression levels after 10 days of drought treatment (Wei et al., 2014). In W14 (*Manihot esculenta* ssp. *flabellifolia*) subspecies, an ancestor of the wild cassava subspecies with strong drought tolerance, 20% of *MeMAPK* genes in leaves and 70% in roots were found to be induced by drought stress (Yan et al., 2016). The high ratio of drought-induced *MeMAPK* genes in roots indicates that MAPK genes may play a regulatory role in water uptake from soil by roots and may help maintain a strong tolerance to drought stress (Yan et al., 2016). When grapevine plants were subjected to drought stress, the expression levels of almost all *VviMAPKKK* genes significantly increased 8 days after drought treatment (Wang et al., 2014). *VviMAPKKK22*, *VviMAPKKK23*, *VviMAPKKK51* and *VviMAPKKK54* transcripts showed greater than 20-fold increased expression. This study provided the first insight into the possible involvement of grapevine MAPKKKs in drought stress (Wang et al., 2014). These results indicated that MAPK cascades play important roles in response to drought stress in horticultural plants.

### MAPK Cascades Involved in Response to Extreme Temperature Stress

Temperature as an important environmental factor has an increasingly significant effect on plant growth and development (Quint et al., 2016). When plants suffer abnormal temperature, the cells present with dehydration, the intracellular pH and osmotic pressure increase, the plasma membrane system and cell structure are damaged, and the functions of organelles such as chloroplasts and mitochondria are abnormal, ultimately leading to metabolic disorders and causing significant losses in plant productivity (Yamazaki et al., 2009; Yin et al., 2014; Lohani et al., 2020). In model plants, MAPK cascades play an important role in response to temperature stress. The MAPK cascade in *Brachypodium distachyon* was temperature sensitive: 90% of the examined MAPK cascade genes were induced under cold stress, and 60% of the genes were induced by high temperature stress in *B. distachyon* (Jiang et al., 2015). In horticultural plants, *SlMPK1* and *SlMPK2*, which are two MAPK genes in tomato (*Solanum lycopersicum*), are involved in brassinosteroid-mediated oxidative and heat stresses (Nie et al., 2013). *SlMPK1* is a negative regulator of thermotolerance in tomato plants. Silencing of *SlMPK1* in transgenic tomato enhances high-temperature tolerance, whereas *SlMPK1*-overexpression transgenic lines displayed lower tolerance to high temperature, with low levels of antioxidative enzyme activities and high levels of H_2_O_2_ and MDA (Ding et al., 2018). *SlMPK3* is a low-temperature stress response gene. Transgenic plants overexpressing *SlMPK3* exhibited a higher seed germination rate and longer root length than wild-type plants. Overexpression of *SlMPK3* increased the activity of antioxidant enzymes, elevated the intracellular levels of proline and soluble sugars, and enhanced plant resistance under cold stress conditions (Yu et al., 2015). In cucumber, most of the MAPK cascade genes could be induced by extreme temperature treatment. Most of the examined *CsMAPKs* (except for *CsMPK3* and *CsMPK7*) were upregulated, and the transcripts of *CsMKK4* exhibited a pronounced increase at 8 h after heat treatment (Wang et al., 2015). In *Fragaria vesca*, the expression of 17 of the 19 MAPK genes (*FvMAPK1*-*12*; *FvMPKK1*-*7*) increased significantly at 18 days after flowering during low-temperature treatment, while the transcript levels of *FvMAPK3*, *FvMPKK1*, *FvMPKK3*, *FvMPKK6* and *FvMPKK7* were significantly upregulated by high-temperature treatment (Zhou et al., 2017). Among these genes, *FvMAPKK3* showed specific activation by cold and heat stresses (Zhou et al., 2017). Research results have shown that mulberry MAPK genes also participate in response to extreme temperature. Eight *MnMAPK* genes were significantly induced by 40°C high-temperature treatment (Wei et al., 2014). Among them, *MnMAPK1*, *MnMAPK5*, *MnMAPK6* and *MnMAPK9* were upregulated, and *MnMAPK2*, *MnMAPK3*, *MnMAPK8* and *MnMAPK10* were downregulated. Under low-temperature (4°C) treatment, the expression levels of *MnMAPK1* and *MnMAPK5* were significantly upregulated (Wei et al., 2014). The *CmMPK1*, *CmMPK3.1*, *CmMPK3.2*, *CmMPK4.2*, *CmMPK6*, *CmMPK9.1*, *CmMPK9.2*, *CmMPK13*, *CmMPK16* and *CmMPK18* genes in *Chrysanthemum morifolium* were induced after cold treatment, but the expression levels of *CmMPK1*, *CmMPK3.1*, *CmMPK3.2*, *CmMPK4.2*, *CmMPK9.1*, *CmMPK9.2*, *CmMPK16* and *CmMPK18* were decreased or remained unchanged after heat shock treatment for 1 h (Song et al., 2018). The available results indicated that MAPK cascades regulated tolerance to heat or cold stresses in horticultural plants.

### MAPK Cascades Involved in Response to Salt Stress

Salinity, as the major threat to agricultural production, endangers more than 50% of irrigated lands worldwide (Hasegawa et al., 2000; Yamaguchi and Blumwald, 2005; Munns et al., 2020). Exposure of plants to salt stress leads to potential disruption of membranes and proteins accompanied by rising levels of reactive oxygen species (ROS) and ultimately results in growth inhibition and loss of crop yields (Krasensky and Jonak, 2012; Yang and Guo, 2018; Osthoff et al., 2019). Li et al. (2015) reported that activation of MAPK protein (recognized by a phosphospecific antibody (pTEpY) and the band between 44 kD and 47 kD) can promote the expression levels of V-H^+^-ATPase, leading to high tolerance to salt in Keyuan-1 peppermint (a salt-tolerant peppermint species). Recently, research showed that this MAPK protein exhibited time-dependent activation in Keyuan-1 peppermint during 12 days of treatment with 150 mM NaCl and primarily modulated the pathway of essential oil metabolism at the transcript and enzyme levels of salt-tolerant peppermint upon NaCl stress (Li et al., 2016). Fen Jiao banana (*Musa* ABB PisangAwak, FJ) has higher tolerance to abiotic stress than BaXi Jiao banana (*Musa acuminate* L. AAA group cv. Cavendish, BX) (Wang et al., 2017). Research results indicated that the ratio of *MAPKK* and *MAPKKK* genes upregulated by salt stress was higher in FJ than in BX, implying that the MAPK cascade may be more active in FJ than in BX in response to salt stress (Wang et al., 2017). In mulberry, *MnMAPKs* can be induced by salt stress. After high-salinity treatment, three *MnMAPKs* (*MnMAPK1*, *MnMAPK9* and *MnMAPK10*) were significantly upregulated, and four *MnMAPKs* (*MnMAPK3*, *MnMAPK4*, *MnMAPK7* and *MnMAPK8*) were significantly downregulated (Wei et al., 2014). In *Chrysanthemum morifolium*, *CmMPK13* and *CmMKK4* were induced by salt stress; they were specifically expressed in roots, and their expression was significantly increased after PEG or salt treatment (Song et al., 2018). In addition, the expression levels of *CmMPK4.2* and *CmMKK2* increased after high-salinity and PEG treatment, which were also shown to interact strongly in yeast. Therefore, CmMKK4-CmMPK13 and CmMKK2-CmMPK4 may be involved in regulating salt tolerance in *C. morifolium* (Song et al., 2018). In cassava, MAPK family genes might be positively or negatively involved in the salt stress response. With high-salinity treatment, *MeMAPK4* was obviously inhibited at all treatment time points, and *MeMAPK16*, *MeMAPK17* and *MeMAPK19* were repressed at several treatment time points. *MeMAPK1* showed upregulation at all treatment time points (Yan et al., 2016). In kiwifruit, with high-salinity treatment, the expression of *AcMAPK4*, *AcMAPK5*, *AcMAPK9* and *AcMAPK12* was significantly upregulated at all treatment time points, indicating that these genes might be important regulators in response to salt stress (Wang G. et al., 2018). Furthermore, in response to salt stress, the expression of five *FvMAPK* genes (*FvMAPK5*, *FvMAPK9*, *FvMAPK10*, *FvMAPK11* and *FvMAPK12*) and three *FvMAPKK* genes (*FvMAPKK1*, *FvMAPKK3* and *FvMAPKK5*) also increased in *F. vesca*, and the transcript levels of *FvMAPKK3* in leaves were specifically activated by salt stress (Zhou et al., 2017). Mounting evidence has shown that MAPK cascades were the key regulator of the response to salt stress in horticultural plants.

## The Function of MAPK Cascades in Responses to Biotic Stresses in Horticultural Plants

During their growth and development, plants are often attacked by bacteria, fungi and viruses. With long-term evolution, higher plants have formed a series of defense mechanisms to resist pathogen infection, such as programmed cell death, cell wall thickening, ROS accumulation, pathogenesis-related (PR) protein synthesis, and transcriptional activation of defense genes (Nejat and Mantri, 2017; Vaahtera et al., 2019). The MAPK cascade is known to be one of the earliest activated pathways during defense activation in response to pathogenic infection (Bi and Zhou, 2017). MAPK cascades are involved in multiple defense responses, including the signaling of plant defense hormones, ROS generation, defense gene activation, and hypersensitive response (HR) cell death (Meng and Zhang, 2013). A comparative transcriptomic analysis was performed using root tissues of equivalent developmental stages between apple replant disease-tolerant Geneva^®^ 935 (G.935) and susceptible Bud 9 (B.9) apple rootstocks after *Pythium ultimum* inoculation. A *mitogen-activated protein kinase kinase kinase 3-like* (MDP0000187103) gene demonstrated specific suppression in B.9, whereas the same gene was consistently upregulated in G.935 (Zhu et al., 2019). *MAPK1*, which belongs to group A MAPKs, played an important role in the defense response to two citrus canker pathogens, *Xanthomonas citri* and *X. aurantifolii*, in citrus (de Oliveira et al., 2013). Increased expression of *MAPK1* was correlated with a reduction in canker symptoms and a decrease in bacterial growth. Overexpression of *MAPK1* in sweet orange resulted in higher transcript levels of defense-related genes and significant accumulation of hydrogen peroxide in response to *X. citri* infection (de Oliveira et al., 2013). Previous studies showed that *SlMPK1*, *SlMPK2* and *SlMPK3* played important roles in the systemin-mediated response to insect herbivory by regulating jasmonic acid (JA) biosynthesis and the expression of JA-dependent defense genes in tomato (Kandoth et al., 2007). *SlMPK* genes were also involved in the Cf-4-mediated HR that mediated plant resistance to *Cladosporium fulvum* (Stulemeijer et al., 2007). Furthermore, *SlMPK2* and *SlMPK3* participated in the defense against *Xanthomonas campestris* pv. *vesicatoria* (Mayrose et al., 2004; Melech-Bonfil and Sessa, 2011). SlMKK2 and SlMKK4, two tomato MAPKKs, were found to activate SlMPK1 and SlMPK2 *in vitro* and to induce cell death when overexpressed in tomato leaves, thus indicating a possible MAPK cascade in the *Pto*-mediated defense response against *Pseudomonas syringae* pv. *tomato* (Pedley and Martin, 2004). SlMAPKKKϵ is required for HR-induced cell death and disease resistance against gram-negative bacterial pathogens in tomato by mediating the SlMAPKKKϵ-MEK2-WIPK/SIPK cascade. Silencing of *SlMAPKKKϵ* compromised tomato resistance to *X. campestris* and *P. syringae* strains, resulting in the appearance of disease symptoms and enhanced bacterial growth (Melech-Bonfil and Sessa, 2010). The triple kinase SlMAPKKKα has been demonstrated to function as a positive regulator of *Pto*-mediated cell death in transgenic *Nicotiana benthamiana* lines (del Pozo et al., 2004). In addition, two MAPK cascades, MEK2-WIPK and MEK1-NTF6, were involved in *Pto*-mediated disease resistance in tomato by regulating the expression of *NPR1*, a key regulator of systemic acquired resistance (Ekengren et al., 2003). Powdery mildew caused by the biotrophic ascomycete *Erysiphe necator* Schw. adversely affects grapevine growth, berry quality and grape production (Fung et al., 2008). When the grape was infected by *E. necator*, a strong increase in the transcripts of most *MAPKKK* genes (*VviMAPKKK46*, *VviMAPKKK50*, *VviMAPKKK31*, *VviMAPKKK32*, *VviMAPKKK39*, *VviMAPKKK38* and *VviMAPKKK34*) was caused, and in particular, *VviMAPKKK50* showed the highest transcript abundance. A few MAPKKK genes (*VviMAPKKK4*, *VviMAPKKK54* and *VviMAPKKK51*) were significantly downregulated by powdery mildew infection, especially *VviMAPKKK54* (Wang et al., 2014). In cucumber, a *Trichoderma*-induced MAPK (TIPK) is involved in fungal defense responses (Shoresh et al., 2006). Furthermore, qRT-PCR analyses were used to examine the expression levels of the *CsMAPK* genes in response to *Pseudoperonospora cubensis*. The results showed that all the examined *CsMAPKs* were downregulated after *P. cubensis* treatment, and the expression levels of *CsMAPKKs* irregularly increased or decreased following *P. cubensis* treatment (Wang et al., 2015). MEKK1-MKK4/5-MPK3/6-WRKY22/29 and MKK9-mediated modules were involved in the defense response to *Plasmodiophora brassicae* in *Brassica rapa*. Three pair-wise genes (*BraMKK4-1/4-2*, *BraMKK5-1/5-2*, and *BraMPK6-1/6-2*) and *BraMPKs* (*MPK3* and *MPK4*) were strongly and continuously activated in the roots of the CS BJN3-2 plants (Chinese cabbage near-isogenic lines (NILs) carrying the clubroot-susceptible allele of *crbcrb*) (Piao et al., 2018). In the modules of MKK9-MPK1/2-WRKY53, MKK9-MPK5 and MKK9-MPK9/19/20, the transcripts of *BraMKK9* and *BraMPK1*, *BraMPK2*, *BraMPK5*, *BraMPK9*, *BraMPK19* and *BraMPK20* were increased in *B. rapa* after *P. brassicae* infection (Piao et al., 2018). MAPK cascades serve as convergence points downstream of multiple cell surface-resident receptors (Devendrakumar et al., 2018). The StMEK2-mediated MAPK cascade is involved in potato immunity dependent on StLRPK1, which is a putative leucine-rich repeat transmembrane receptor-like kinase (Wang H. et al., 2018). Silencing *StMEK2* in *StLRRK1*-overexpressing *N. benthamiana* plants attenuates resistance to *Phytophthora infestans* (Wang H. et al., 2018). Based on the previous research, we noticed that MAPK cascades regulate the disease resistance of horticultural plants through multiple signal transduction pathways.

## Conclusion

Mounting evidence indicates that the plant stress-resistance signal transduction process is a complex network system, and that the MAPK cascade is at the center of the network. Through phosphorylation and dephosphorylation, the MAPK cascade progressively amplifies and transmits a variety of stress signals to downstream response factors and causes a series of stress responses. Analyzing the members of the MAPK cascade and its mechanism are critical for improving horticultural crop resistance through molecular biology. Furthermore, deeper knowledge of the mechanism of MAPK cascades might facilitate the development of novel strategies to improve stress tolerance in horticultural plants (Šamajová et al., 2013). Genetic engineering techniques offer various applications for improvement of biotic and abiotic stress tolerance in horticultural crops (Nehanjali et al., 2017). MAPK cascades, as regulators of gene transcription with central roles in signal transduction, have already been employed to increase abiotic stress tolerance (Nehanjali et al., 2017).

Although a large number of studies in horticultural plants have shown that MAPK cascades are involved in multiple biological processes in responses to abiotic and biotic stresses, research on the function of MAPK genes or the mechanism by which the MAPK cascade regulates plant stress resistance is still limited. In addition, different stress stimuli can activate the same MAPK cascade genes. For example, in strawberry, drought and salt damage can both activate MAPK5 (Zhou et al., 2017). Temperature and pathogens can activate *MAPK1*-*3* genes in tomato (Kandoth et al., 2007; Nie et al., 2013; Yu et al., 2015; Ding et al., 2018). The same stress stimuli can also activate different MAPK cascades; for example, *P. brassicae* can activate MKK9-MPK1/2-WRKY53, MKK9-MPK5 and MKK9-MPK9/19/20 in *B. rapa* (Piao et al., 2018). Therefore, how the same MAPK cascade is activated by different stresses and causes different responses and how different MAPK cascades coordinate the division of labor under the same stress remain to be further verified by researchers. Thus, further analyses of MAPK cascades and the molecular mechanisms of plant stress resistance have great significance for elucidating the entire stress-tolerance signal transduction pathway in horticultural plants.

## Author Contributions

CheW and LL conceived the project. CheW and XH wrote the article. XH, ChuW, and HW performed the bioinformatics analysis.

## Funding

This work was financially supported by the Natural Science Foundation of Shandong Province (Grant No. ZR2019BC015) and the Agricultural Scientific and Technological Innovation Project of Shandong Academy of Agricultural Sciences (Grant Nos. CXGC2018E22 and CXGC2018F03).

## Conflict of Interest

The authors declare that the research was conducted in the absence of any commercial or financial relationships that could be construed as a potential conflict of interest.

## References

[B1] Bai Y. Kissoudis C. Yan Z. Visser R. G. F. van der Linden G. (2018). Plant behaviour under combined stress: tomato responses to combined salinity and pathogen stress. Plant J. 93, 781–793. 10.1111/tpj.13800 29237240

[B2] Bi G. Zhou J. M. (2017). MAP kinase signaling pathways: a hub of plant-microbe interactions. Cell Host. Microbe 21, 270–273. 10.1016/j.chom.2017.02.004 28279328

[B3] Çakır B. Kılıçkaya O. (2015). Mitogen-activated protein kinase cascades in *Vitis vinifera.* Front. Plant Sci. 6, 556–574. 10.3389/fpls.2015.00556 26257761 PMC4511077

[B4] Colcombet J. Hirt H. (2008). Arabidopsis MAPKs: a complex signalling network involved in multiple biological processes. Biochem. J. 413, 217–226. 10.1042/BJ20080625 18570633

[B5] de Oliveira M. L. Silva C. C. L. Abe V. Y. Costa M. G. Cernadas R. A. Benedetti C. E. (2013). Increased resistance against citrus canker mediated by a citrus mitogen-activated protein kinase. Mol. Plant Microbe Interact. 26, 1190–1199. 10.1094/MPMI-04-13-0122-R 23777433

[B6] del Pozo O. Pedley K. F. Martin G. B. (2004). MAPKKKalpha is a positive regulator of cell death associated with both plant immunity and disease. EMBO J. 23, 3072–3082. 10.1038/sj.emboj.7600283 15272302 PMC514913

[B7] Devendrakumar K. T. Li X. Zhang Y. (2018). MAP kinase signalling: interplays between plant PAMP- and effector-triggered immunity. Cell. Mol. Life Sci. 75, 2981–2989. 10.1007/s00018-018-2839-3 29789867 PMC11105241

[B8] Ding H. He J. Wu Y. Wu X. Ge C. Wang Y. (2018). The tomato mitogen-activated protein kinase SlMPK1 Is as a negative regulator of the high-temperature stress response. Plant Physiol. 177, 633–651. 10.1104/pp.18.00067 29678861 PMC6001329

[B9] Ekengren S. K. Liu Y. Schiff M. Dinesh-Kumar S. P. Martin G. B. (2003). Two MAPK cascades, NPR1, and TGA transcription factors play a role in Pto-mediated disease resistance in tomato. Plant J. 36, 905–917. 10.1046/j.1365-313X.2003.01944.x 14675454

[B10] Fung R. W. Gonzalo M. Fekete C. Kovacs L. G. He Y. Marsh E. (2008). Powdery mildew induces defense-oriented reprogramming of the transcriptome in a susceptible but not in a resistant grapevine. Plant Physiol. 146, 236–249. 10.1104/pp.107.108712 17993546 PMC2230561

[B11] Hamel L. P. Nicole M. C. Sritubtim S. Morency M. J. Ellis M. Ehlting J. (2006). Ancient signals: comparative genomics of plant MAPK and MAPKK gene families. Trends Plant Sci. 11, 192–198. 10.1016/j.tplants.2006.02.007 16537113

[B12] Hasegawa P. M. Bressan R. A. Zhu J. K. Bohnert H. J. (2000). Plant cellular and molecular responses to high salinity. Annu. Rev. Plant Physiol. Plant Mol. Biol. 51, 463–499. 10.1146/annurev.arplant.51.1.463 15012199

[B13] Huang X. S. Luo T. Fu X. Z. Fan Q. J. Liu J. H. (2011). Cloning and molecular characterization of a mitogen-activated protein kinase gene from *Poncirus trifoliata* whose ectopic expression confers dehydration/drought tolerance in transgenic tobacco. J. Exp. Bot. 62, 5191–5206. 10.1093/jxb/err229 21778184 PMC3193021

[B14] Huang D. Ma M. N. Wang Q. Zhang M. X. Jing G. Q. Li C. (2020). Arbuscular mycorrhizal fungi enhanced drought resistance in apple by regulating genes in the MAPK pathway. Plant Physiol. Biochem. 149, 245–255. 10.1016/j.plaphy.2020.02.020 32087536

[B15] Ichimura K. Shinozaki K. Tena G. Sheen J. Henry Y. Champion A. (2002). Mitogen-activated protein kinase cascades in plants: a new nomenclature. Trends Plant Sci. 7, 301–308. 10.1016/S1360-1385(02)02302-6 12119167

[B16] Ji T. Li S. Huang M. Di Q. Wang X. Wei M. (2017). Overexpression of cucumber phospholipase D alpha gene (CsPLDalpha) in tobacco enhanced salinity stress tolerance by regulating Na(+)-K(+) balance and lipid peroxidation. Front. Plant Sci. 8, 499. 10.3389/fpls.2017.00499 28439282 PMC5383712

[B17] Jiang M. Wen F. Cao J. Li P. She J. Chu Z. (2015). Genome-wide exploration of the molecular evolution and regulatory network of mitogen-activated protein kinase cascades upon multiple stresses in *Brachypodium distachyon* . BMC Genomics 16, 228. 10.1186/s12864-015-1452-1 25886731 PMC4404688

[B18] Jonak C. Okresz L. Bogre L. Hirt H. (2002). Complexity, cross talk and integration of plant MAP kinase signalling. Curr. Opin. Plant Biol. 5, 415–424. 10.1016/S1369-5266(02)00285-6 12183180

[B19] Kandoth P. K. Ranf S. Pancholi S. S. Jayanty S. Walla M. D. Miller W. (2007). Tomato MAPKs LeMPK1, LeMPK2, and LeMPK3 function in the systemin-mediated defense response against herbivorous insects. Proc. Natl. Acad. Sci. U. S. A. 104, 12205–12210. 10.1073/pnas.0700344104 17623784 PMC1924534

[B20] Kissoudis C. van de Wiel C. Visser R. G. van der Linden G. (2014). Enhancing crop resilience to combined abiotic and biotic stress through the dissection of physiological and molecular crosstalk. Front. Plant Sci. 5, 207. 10.3389/fpls.2014.00207 24904607 PMC4032886

[B21] Komis G. Šamajová O. Ovečka M. Šamaj J. (2018). Cell and developmental biology of plant mitogen-activated protein kinases. Annu. Rev. Plant Biol. 69, 237–265. 10.1146/annurev-arplant-042817-040314 29489398

[B22] Kong F. Wang J. Cheng L. Liu S. Wu J. Peng Z. (2012). Genome-wide analysis of the mitogen-activated protein kinase gene family in *Solanum lycopersicum* . Gene 499, 108–120. 10.1016/j.gene.2012.01.048 22306326

[B23] Krasensky J. Jonak C. (2012). Drought, salt, and temperature stress-induced metabolic rearrangements and regulatory networks. J. Exp. Bot. 63, 1593–1608. 10.1093/jxb/err460 22291134 PMC4359903

[B24] Li Z. Zhen Z. Guo K. Harvey P. Li J. Yang H. (2015). MAPK-mediated enhanced expression of vacuolar H(+)-ATPase confers the improved adaption to NaCl stress in a halotolerate peppermint (*Mentha piperita* L.). Protoplasma 253, 553–569. 10.1007/s00709-015-0834-1 25999237

[B25] Li Z. Wang W. Li G. Guo K. Harvey P. Chen Q. (2016). MAPK-mediated regulation of growth and essential oil composition in a salt-tolerant peppermint (*Mentha piperita* L.) under NaCl stress. Protoplasma 253, 1541–1556. 10.1007/s00709-015-0915-1 26631016

[B26] Lohani N. Singh M. B. Bhalla P. L. (2020). High temperature susceptibility of sexual reproduction in crop plants. J. Exp. Bot. 71, 555–568. 10.1093/jxb/erz426 31560053

[B27] Mayrose M. Bonshtien A. Sessa G. (2004). LeMPK3 is a mitogen-activated protein kinase with dual specificity induced during tomato defense and wounding responses. J. Biol. Chem. 279, 14819–14827. 10.1074/jbc.M313388200 14742423

[B28] Melech-Bonfil S. Sessa G. (2010). Tomato MAPKKKepsilon is a positive regulator of cell-death signaling networks associated with plant immunity. Plant J. 64, 379–391. 10.1111/j.1365-313X.2010.04333.x 21049563

[B29] Melech-Bonfil S. Sessa G. (2011). The SlMKK2 and SlMPK2 genes play a role in tomato disease resistance to *Xanthomonas campestris* pv. vesicatoria. Plant Signal. Behav. 6, 154–156. 10.4161/psb.6.1.14311 21248478 PMC3122032

[B30] Meng X. Z. Zhang S. Q. (2013). MAPK cascades in plant disease resistance signaling. Annu. Rev. Phytopathol. 51, 245–266. 10.1146/annurev-phyto-082712-102314 23663002

[B31] Meng Y. Ma N. Zhang Q. You Q. Li N. Ali Khan M. (2014). Precise spatio-temporal modulation of ACC synthase by MPK6 cascade mediates the response of rose flowers to rehydration. Plant J. 79, 941–950. 10.1111/tpj.12594 24942184

[B32] Morris P. (2001). MAP kinase signal transduction pathways in plants. New Phytol. 151, 67–89. 10.1046/j.1469-8137.2001.00167.x 33873387

[B33] Munns R. Passioura J. B. Colmer T. D. Byrt C. S. (2020). Osmotic adjustment and energy limitations to plant growth in saline soil. New Phytol. 225, 1091–1096. 10.1111/nph.15862 31006123

[B34] Nehanjali P. Kunwar H. S. Deepika S. Lal S. Pankaj K. Nanjundan J. (2017). Genetic engineering strategies for biotic and abiotic stress tolerance and quality enhancement in horticultural crops: a comprehensive review. 3 Biotech. 7 (4), 239. 10.1007/s13205-017-0870-y PMC5507805 28702937

[B35] Nejat N. Mantri N. (2017). Plant immune system: crosstalk between responses to biotic and abiotic stresses the missing link in understanding plant defence. Curr. Issues Mol. Biol. 23, 1–16. 10.21775/cimb.023.001 28154243

[B36] Nie W. F. Wang M. M. Xia X. J. Zhou Y. H. Shi K. Chen Z. (2013). Silencing of tomato RBOH1 and MPK2 abolishes brassinosteroid-induced H_2_O_2_ generation and stress tolerance. Plant Cell Environ. 36, 789–803. 10.1111/pce.12014 22994632

[B37] Osthoff A. Rose P. D. D. Baldauf J. A. Piepho H. P. Hochholdinger F. (2019). Transcriptomic reprogramming of barley seminal roots by combined water deficit and salt stress. BMC Genomics 20, 325. 10.1186/s12864-019-5634-0 31035922 PMC6489292

[B38] Pedley K. F. Martin G. B. (2004). Identification of MAPKs and their possible MAPK kinase activators involved in the Pto-mediated defense response of tomato. J. Biol. Chem. 279, 49229–49235. 10.1074/jbc.M410323200 15371431

[B39] Peng L. X. Gu L. K. Zheng C. C. Li D. Q. Shu H. R. (2006). Expression of MaMAPK gene in seedlings of *Malus* L. under water stress. Acta Biochim. Biophys. Sin. 38, 281–286. 10.1093/abbs/38.4.281 16604268

[B40] Piao Y. Jin K. He Y. Liu J. Liu S. Li X. (2018). Genome-wide identification and role of MKK and MPK gene families in clubroot resistance of *Brassica rapa* . PloS One 13, e0191015. 10.1371/journal.pone.0191015 29444111 PMC5812557

[B41] Quint M. Delker C. Franklin K. A. Wigge P. A. Halliday K. J. van Zanten M. (2016). Molecular and genetic control of plant thermomorphogenesis. Nat. Plants 2, 15190. 10.1038/nplants.2015.190 27250752

[B42] Ramani S. Chelliah J. (2007). UV-B-induced signaling events leading to enhanced-production of catharanthine in *Catharanthus roseus* cell suspension cultures. BMC Plant Biol. 7, 61. 10.1186/1471-2229-7-61 17988378 PMC2213653

[B43] Rao K. P. Richa T. Kumar K. Raghuram B. Sinha A. K. (2010). In silico analysis reveals 75 members of mitogen-activated protein kinase kinase kinase gene family in rice. DNA Res. 17, 139–153. 10.1093/dnares/dsq011 20395279 PMC2885274

[B44] Rodriguez M. C. Petersen M. Mundy J. (2010). Mitogen-activated protein kinase signaling in plants. Annu. Rev. Plant Biol. 61, 621–649. 10.1146/annurev-arplant-042809-112252 20441529

[B45] Šamajová O. Plíhal O. Al-Yousif M. Hirt H. Šamaj J. (2013). Improvement of stress tolerance in plants by genetic manipulation of Mitogen-activated protein kinases. Biotechnol. Adv. 31, 118–128. 10.1016/j.biotechadv.2011.12.002 22198202

[B46] Shi J. Zhang L. An H. Wu C. Guo X. (2011). GhMPK16, a novel stress-responsive group D MAPK gene from cotton, is involved in disease resistance and drought sensitivity. BMC Mol. Biol. 12, 22. 10.1186/1471-2199-12-22 21575189 PMC3117701

[B47] Shoresh M. Gal-On A. Leibman D. Chet I. (2006). Characterization of a mitogen-activated protein kinase gene from cucumber required for trichoderma-conferred plant resistance. Plant Physiol. 142, 1169–1179. 10.1104/pp.106.082107 16950863 PMC1630744

[B48] Shulaev V. Sargent D. J. Crowhurst R. N. Mockler T. C. Folkerts O. Delcher A. L. (2011). The genome of woodland strawberry (*Fragaria vesca*). Nat. Genet. 43, 109–116. 10.1038/ng.740 21186353 PMC3326587

[B49] Song A. Hu Y. Ding L. Zhang X. Li P. Liu Y. (2018). Comprehensive analysis of mitogen-activated protein kinase cascades in chrysanthemum. Peer J. 6, e5037. 10.7717/peerj.5037 29942696 PMC6014330

[B50] Stulemeijer I. J. E. Stratmann J. W. Joosten M. H. A. J. (2007). Tomato mitogen-activated protein kinases LeMPK1, LeMPK2, and LeMPK3 are activated during the Cf-4/Avr4-induced hypersensitive response and have distinct phosphorylation specificities. Plant Physiol. 144, 1481–1494. 10.1104/pp.107.101063 17478632 PMC1914120

[B51] Sun M. Xu Y. Huang J. Jiang Z. Shu H. Wang H. (2017). Global identification, classification, and expression analysis of MAPKKK genes: functional characterization of mdraf5 reveals evolution and drought-responsive profile in apple. Sci. Rep. 7, 13511. 10.1038/s41598-017-13627-2 29044159 PMC5647345

[B52] Taj G. Agarwal P. Grant M. Kumar A. (2010). MAPK machinery in plants: recognition and response to different stresses through multiple signal transduction pathways. Plant Signal. Behav. 5, 1370–1378. 10.4161/psb.5.11.13020 20980831 PMC3115236

[B53] Vaahtera L. Schulz J. Hamann T. (2019). Cell wall integrity maintenance during plant development and interaction with the environment. Nat. Plants 5, 924–932. 10.1038/s41477-019-0502-0 31506641

[B54] Wang G. Lovato A. Polverari A. Wang M. Liang Y. H. Ma Y. C. (2014). Genome-wide identification and analysis of mitogen activated protein kinase kinase kinase gene family in grapevine (*Vitis vinifera*). BMC Plant Biol. 14, 219. 10.1186/s12870-014-0219-1 25158790 PMC4243721

[B55] Wang J. Pan C. Wang Y. Ye L. Wu J. Chen L. (2015). Genome-wide identification of MAPK, MAPKK, and MAPKKK gene families and transcriptional profiling analysis during development and stress response in cucumber. BMC Genomics 16, 386. 10.1186/s12864-015-1621-2 25976104 PMC4432876

[B56] Wang L. Hu W. Tie W. Ding Z. Ding X. Liu Y. (2017). The MAPKKK and MAPKK gene families in banana: identification, phylogeny and expression during development, ripening and abiotic stress. Sci. Rep. 7, 1159. 10.1038/s41598-017-01357-4 28442729 PMC5430750

[B57] Wang G. Wang T. Jia Z.-H. Xuan J.-P. Pan D. L. Guo Z. R. (2018). Genome-wide bioinformatics analysis of MAPK gene family in Kiwifruit (*Actinidia Chinensis*). Int. J. Mol. Sci. 19, E2510. 10.3390/ijms19092510 30149559 PMC6164783

[B58] Wang H. Chen Y. Wu X. Long Z. Sun C. Wang H. (2018). A potato STRUBBELIG-RECEPTOR FAMILY member, StLRPK1, associates with StSERK3A/BAK1 and activates immunity. J. Exp. Bot. 69, 5573–5586. 10.1093/jxb/ery310 30137408 PMC6255708

[B59] Wang C. Guo H. He X. Zhang S. Wang J. Wang L. (2019). Scaffold protein GhMORG1 enhances the resistance of cotton to Fusarium oxysporum by facilitating the MKK6-MPK4 cascade. Plant Biotechnol. J. 18, 1421–1433. 10.1111/pbi.13307 31794094 PMC7206998

[B60] Wei C. Liu X. Long D. Guo Q. Fang Y. Bian C. (2014). Molecular cloning and expression analysis of mulberry MAPK gene family. Plant Physiol. Biochem. 77, 108–116. 10.1016/j.plaphy.2014.02.002 24583344

[B61] Weng C.-M. Lu J.-X. Wan H.-F. Wang S.-W. Wang Z. Lu K. (2014). Over-expression of BnMAPK1 in Brassica napus enhances tolerance to drought stress. J. Integr. Agric. 13, 2407–2415. 10.1016/S2095-3119(13)60696-6

[B62] Wu J. Wang J. Pan C. Guan X. Wang Y. Liu S. (2014). Genome-wide identification of MAPKK and MAPKKK gene families in tomato and transcriptional profiling analysis during development and stress response. PloS One 9, e103032. 10.1371/journal.pone.0103032 25036993 PMC4103895

[B63] Xu J. Zhang S. (2015). Mitogen-activated protein kinase cascades in signaling plant growth and development. Trends Plant Sci. 20, 56–64. 10.1016/j.tplants.2014.10.001 25457109

[B64] Yamaguchi T. Blumwald E. (2005). Developing salt-tolerant crop plants: challenges and opportunities. Trends Plant Sci. 10, 615–620. 10.1016/j.tplants.2005.10.002 16280254

[B65] Yamazaki T. Kawamura Y. Uemura M. (2009). Extracellular freezing-induced mechanical stress and surface area regulation on the plasma membrane in cold-acclimated plant cells. Plant Signal. Behav. 4, 231–233. 10.4161/psb.4.3.7911 19721759 PMC2652538

[B66] Yan Y. Wang L. Ding Z. Tie W. Ding X. Zeng C. (2016). Genome-wide identification and expression analysis of the mitogen-activated protein kinase gene family in cassava. Front. Plant Sci. 7, 1294. 10.3389/fpls.2016.01294 27625666 PMC5003926

[B67] Yanagawa Y. Yoda H. Osaki K. Amano Y. Aono M. Seo S. (2016). Mitogen-activated protein kinase 4-like carrying an MEY motif instead of a TXY motif is involved in ozone tolerance and regulation of stomatal closure in tobacco. J. Exp. Bot. 67, 3471–3479. 10.1093/jxb/erw173 27126796 PMC4892734

[B68] Yang Y. Guo Y. (2018). Elucidating the molecular mechanisms mediating plant salt-stress responses. New Phytol. 217, 523–539. 10.1111/nph.14920 29205383

[B69] Yin Y.-X. Guo W.-L. Zhang Y.-L. Ji J.-J. Xiao H.-J. Yan F. (2014). Cloning and characterisation of a pepper aquaporin, CaAQP, which reduces chilling stress in transgenic tobacco plants. Plant Cell Tissue Organ Cult. 118, 431–444. 10.1007/s11240-014-0495-3

[B70] Yu L. Yan J. Yang Y. Zhu W. (2015). Overexpression of tomato mitogen-activated protein kinase SlMPK3 in tobacco increases tolerance to low temperature stress. Plant Cell Tissue Organ Cult. 121, 21–34. 10.1007/s11240-014-0675-1

[B71] Zandalinas S. I. Fritschi F. B. Mittler R. (2019). Signal transduction networks during stress combination. J. Exp. Bot. 71, 1734–1741. 10.1093/jxb/erz486 31665392

[B72] Zhang S. Klessig D. F. (2001). MAPK cascades in plant defense signaling. Trends Plant Sci. 6, 520–527. 10.1016/S1360-1385(01)02103-3 11701380

[B73] Zhang S. Xu R. Luo X. Jiang Z. Shu H. (2013). Genome-wide identification and expression analysis of MAPK and MAPKK gene family in *Malus* domestica. Gene 531, 377–387. 10.1016/j.gene.2013.07.107 23939467

[B74] Zhang H. Li Y. Zhu J.-K. (2018). Developing naturally stress-resistant crops for a sustainable agriculture. Nat. Plants 4, 989–996. 10.1038/s41477-018-0309-4 30478360

[B75] Zhang M. Su J. Zhang Y. Xu J. Zhang S. (2018). Conveying endogenous and exogenous signals: MAPK cascades in plant growth and defense. Curr. Opin. Plant Biol. 45, 1–10. 10.1016/j.pbi.2018.04.012 29753266

[B76] Zhou J. Xia X. J. Zhou Y. H. Shi K. Chen Z. Yu J. Q. (2014). RBOH1-dependent H2O2 production and subsequent activation of MPK1/2 play an important role in acclimation-induced cross-tolerance in tomato. J. Exp. Bot. 65, 595–607. 10.1093/jxb/ert404 24323505 PMC3904713

[B77] Zhou H. Ren S. Han Y. Zhang Q. Qin L. Xing Y. (2017). Identification and analysis of mitogen-activated protein kinase (MAPK) cascades in *Fragaria vesca* . Int. J. Mol. Sci. 18, E1766. 10.3390/ijms18081766 28805715 PMC5578155

[B78] Zhu Y. Shao J. Zhou Z. Davis R. E. (2019). Genotype-specific suppression of multiple defense pathways in apple root during infection by *Pythium ultimum* . Hortic. Res. 6, 10. 10.1038/s41438-018-0087-1 30603095 PMC6312547

[B79] Zhu J. K. (2016). Abiotic stress signaling and responses in plants. Cell 167, 313–324. 10.1016/j.cell.2016.08.029 27716505 PMC5104190

